# Developing Serious Video Games to Treat Attention Deficit Hyperactivity Disorder: Tutorial Guide

**DOI:** 10.2196/33884

**Published:** 2022-08-01

**Authors:** Aarón Sújar, Marina Martín-Moratinos, María Rodrigo-Yanguas, Marcos Bella-Fernández, Carlos González-Tardón, David Delgado-Gómez, Hilario Blasco-Fontecilla

**Affiliations:** 1 Department of Psychiatry Hospital Universitario Puerta de Hierro Majadahonda Majadahonda Spain; 2 Department of Computer Engineering Universidad Rey Juan Carlos Madrid Spain; 3 Faculty of Medicine Universidad Autónoma de Madrid Madrid Spain; 4 Faculty of Psychology Universidad Autónoma de Madrid Madrid Spain; 5 Department of Psychology Universidad Pontificia de Comillas Madrid Spain; 6 Tecnocampus Universitat Pompeu Fabra Mataró Spain; 7 Department of Statistics Universidad Carlos III Leganés Spain; 8 ITA Mental Health Madrid Spain; 9 Centro de Investigación Biomédica en Red Salud Mental Madrid Spain

**Keywords:** serious video games, ADHD, treatment, video games, cognitive, cognitive disorder, games

## Abstract

Video game–based therapeutic interventions have demonstrated some effectiveness in decreasing the symptoms of attention deficit hyperactivity disorder (ADHD). Compared with more traditional strategies within the multimodal treatment of ADHD, video games have certain advantages such as being comfortable, flexible, and cost-efficient. However, establishing the most appropriate type(s) of video games that should be used for this treatment remains a matter of debate, including the commercial existing video games or serious video games that are specifically constructed to target specific disorders. This guide represents a starting point for developing serious video games aimed at treating ADHD. We summarize the key points that need to be addressed to generate an effective and motivating game-based treatment. Following recommendations from the literature to create game-based treatments, we describe the development stages of a serious video game for treating ADHD. Game design should consider the interests of future users; game mechanics should be based on cognitive exercises; and therapeutic mechanisms must include the control of difficulty, engagement, motivation, time constraints, and reinforcement. To elaborate upon this guide, we performed a narrative review focused on the use of video games for the treatment of ADHD, and were inspired by our own experience during the development of the game “The Secret Trail of Moon.”

## Introduction

Attention deficit hyperactivity disorder (ADHD) is the most frequent neurodevelopmental disorder with a worldwide prevalence ranging between 4% and 8% [1]. Core ADHD symptoms (inattention, hyperactivity, and impulsivity) negatively impact social, emotional, and/or cognitive domains compromising two or more areas of daily life [2]. The prognosis is clouded by comorbidities such as executive dysfunction [3] or emotional dysregulation, among others [4]. If undetected or untreated, people with ADHD have a higher probability of accidents, school dropout, addictions, and mortality [5].

According to guidelines, the treatment of choice for ADHD is multimodal [6]. Low adherence to medication is common [7], which is explained by several factors: adverse effects, parent preferences, misuse, and/or omissions [8]. Cognitive behavioral therapy (CBT) is the most effective psychological treatment for ADHD [6]. Although CBT has shown effectiveness in adolescents with ADHD [9], psychotherapies are not usually implemented within public health systems [10]. Furthermore, lack of motivation and engagement are core characteristics of ADHD [4]. Accordingly, any intervention aimed at rehabilitating ADHD should be focused on fun. New technologies, particularly video games, have the potential to fulfill this goal. Indeed, Sonuga-Barke [11] affirms that the playful elements of video games help to maintain motivation for engaging in therapy.

According to the Interactive Software Federation Europe [12], there are currently more than 24 million game players in the age group of 6-15 years across the European market. People with ADHD are more prone to use video games, being up to three times more likely to develop an addiction [13]. However, appropriate use of video games may complement multimodal treatment [14]. Indeed, there is preliminary evidence about the potential use of serious video games to treat ADHD [15-17]. Furthermore, there are guidelines for the creation of health-based games. For example, Duncan et al [18] proposed useful tools (Playbook) for multidisciplinary teams devoted to the development of serious games–based therapies. Kinross [19] proposed a methodology for creating health games inspired by current medical intervention methodologies. Lastly, Baranowski [20] highlights the minimum descriptions that must be considered when developing a new game for health.

The aim of this Tutorial is to provide a basic guide for developers of serious video games targeting ADHD by considering the particularities (eg, lack of adherence, addiction) of this disorder. We offer a roadmap for companies, programmers, and researchers interested in this field. We take advantage of our own experience in developing the serious game “The Secret Trail of Moon” (TSTM) [21-23], a serious video game designed to treat ADHD, which has been tested in a randomized clinical trial (ClinicalTrials.gov NCT04355065).

## Benefits of Video Games

Video games offer intrinsic benefits that can be exploited for other purposes. Some studies stressed that video games could have beneficial cognitive effects on attention and visuospatial abilities in adults [24]; can improve attention, effort, and motivation [25]; or can be effective on top-down attentional control, task-switching, processing speed, and increased time perception [26]. Other studies outline the motivational capacity of video games on emotional and social skills [27].

Video games created with nongame objectives, which are known as “serious video games,” have been developed for different activities such as training, education, or evaluation in nonludic environments [28]. Serious video game activities are motivationally challenging while simultaneously offering users a fun learning experience. Their narratives and technical resources can increase the level of engagement of the user to pursue a specific objective [29]. Levels of engagement are linked to positive emotions produced by effort and overcoming obstacles, which are essential aspects in turning a video game into a tool [30].

Video game–based therapeutic interventions have proven to be useful in health care environments. As previously mentioned, children with ADHD have low intrinsic motivation and poor internal language, which make it difficult for them to complete tasks, leading to procrastination. This low intrinsic motivation causes them to become bored earlier, relying more on an external stimulus or more engaging tasks [4]. Therapeutic interventions based on gamification and video games can be a good strategy for overcoming this problem. In fact, video game–based therapeutic tools appear to be useful and effective in the treatment of ADHD. Engagement rates with video game–based interventions are generally high, with low rates of dropouts (see [15,17] for reviews).

## IDEAL-Games Framework

### Overview

**Figure 1:**
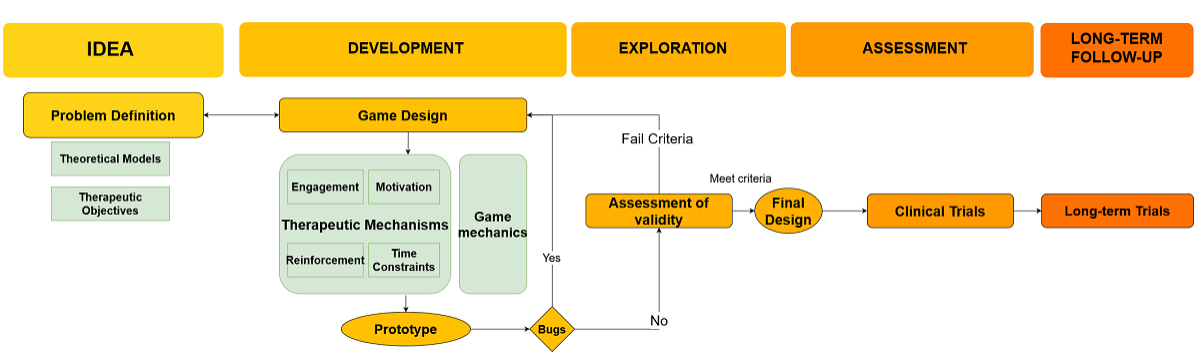
IDEAL-Games framework [22] adapted to create a general guide for game-based attention deficit hyperactivity disorder treatment.

Taking into account the references on the creation of health-based games mentioned in the Introduction, we have adapted Kinross’ [19] proposal and the terminology of Duncan et al [18] and Baranowski [20]. To create a successful therapeutic game, three issues need to be addressed [31]: (1) focus on therapeutic objectives, (2) use game design principles, and (3) incorporate patients’ opinions to adjust to their interests. Figure 1 shows the IDEAL-Games proposal. The IDEAL-Games framework describes the video game development process: (1) Idea, (2) Development, (3) Exploration, (4) Evaluation of the effectiveness, and (5) Long-term assessment to understand the effects over time [19].

Throughout the process, the collaboration of a multidisciplinary team is essential to balance clinical and technical components [31]. Duncan et al [18] remarked that clinicians and researchers master the mechanisms of health behavior change but know much less about the creation of an optimal player experience compared with game developers. Therefore, it is important to “create” a common language and for knowledge to be condensed in a Game Playbook [18] or game design document, eliminating jargon and creating a document that can be easily understood by all members of the team. In the case of TSTM [21], the consortium includes: (1) health care professionals, (2) a serious video game consultant, and (3) a game development company.

### Idea

In the video game industry, a preproduction phase is common. The idea starts by detecting an unmet need. The planning of a project is key to obtain a quality product that is developed within the estimated time and cost. In the case of game-based treatment, the team should stress the main treatment goal, which is based on the literature and the theoretical models chosen. Problem definition determines the final goal. In our example, TSTM [21] was based on the models proposed by Russell Barkley [32] and Thomas Brown [33]. See Rodrigo-Yanguas et al [21,22] for a description of the general characteristics of TSTM.

**Figure 2:**
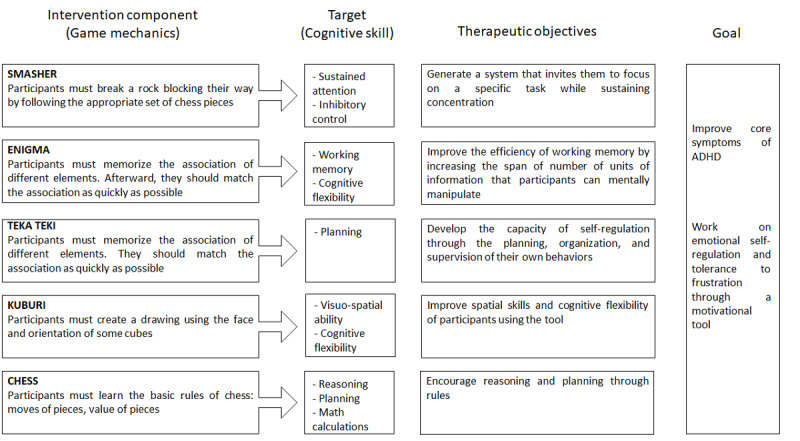
Logic model built for The Secret Trail of Moon (based on Duncan et al [18]). ADHD: attention deficit hyperactivity disorder.

The idea stage is similar to the preproduction of a video game and refers to the process where a multidisciplinary team identifies the therapeutic goals of the video game. This stage provides a clear overview of what the team aims to achieve during the game-play experience and the first drafts of the game that will be developed in the next stage of the process. They should identify the targeted variables aligned with the therapeutic objectives and the strategies that will be followed to achieve the desired outcome. In the case of TSTM, developing these game mechanics involved hours of discussion within the team to find a balance between psychology and technology: the developers wanted a fun game, while the mental health professionals required internal validity of the game based on psychological constructs. Figure 2 displays the logic model built for TSTM.

The use of new technologies may enhance ecological validity through incorporating complexity, novelty, and diversity in the process of developing the video game [34]. However, choosing an innovative technology represents a challenge. The choice will determine both the game and therapeutics mechanics, and affects the entire developmental stage. Developers must consider the barriers of entry, licensing costs, previous experience, and the scalability of the platform chosen. On the patient side, the developer team must consider the commonly used platforms by the general public, the price, and their commercial availability. The inclusion of these factors can enhance the patient’s motivation, isolate the patient from outside distractions, or complement the development of other qualities, which can in turn increase the effectiveness of the treatment. For instance, TSTM uses Playstation VR [22], Benzing and Schmidt [35] used Microsoft Kinect to develop an exergame, and Lim et al [36] developed an attention training game where children play via the signals detected by electroencephalogram electrodes.

Virtual reality (VR) has been a major challenge in the development of TSTM. An example of this is the Kitsune game [21], which was programmed but could not be finally implemented for two reasons: a failed design (Kitsune failed to work on inhibitory control) and motion sickness (due to an interface very close to the player’s vision and being a game in motion). Therefore, we recommend choosing the technology with caution and establishing a good design.

### Development

#### User-Centered Design

In this stage, the multidisciplinary team builds the game’s prototype, considering the game components highlighted by Kinross [19]: game mechanics balanced with elements of game design to improve engagement, motivation, and reinforcement.

Since video games intrinsically have a motivational design [29], a serious video game should be similarly designed. In line with the IDEAL-Games framework, we suggest the user-centered design (UCD) as a design methodology. This iterative methodology is oriented to take into account what the users themselves expect from the game [37]. Finally, compared with commercial games, serious video games must be scientifically tested to check that the usability and efficiency are adequate.

The UCD methodology also allows for recording user performance; collecting this preliminary data with real users allows for closer adjustment to the patient’s needs. In the development of TSTM [21,22], there were two events that emerged in user testing. Enigma had to be refined to improve the impact on working memory, and for Smasher, including a bug representing a distractor (a bird flying in a strange way), psychologists suggested that this incoherent stimulus could be more distracting and was incorporated as an advanced distractor. UCD methodology allowed us to redesign the game mechanics considering how people with ADHD behaved during the game.

The initial TSTM design was modified following suggestions from patients with ADHD about esthetics, game difficulty, or rewards [22]. The UCD methodology fulfilled two additional objectives: the designers checked the correct functioning of the game and the ADHD patients felt involved in the development process.

#### Engaging a Patient With ADHD to Begin Treatment

Treatment with therapeutic video games may help to improve adherence to multimodal treatment in ADHD by bringing patients closer to treatment.

The game’s theme is important in the design process, as player preferences may differ from one player to another. Games such as EndeavorRx [38] or Plan-it-Commander [39] are inspired by space and astronauts. Regarding TSTM, each mini game takes place in the forest, as regular interaction with nature can be beneficial for people with ADHD [40]. Furthermore, it is possible to design exercises that are compatible with different game environments. Thus, users could choose the setting of the game without modifying its mechanics. By doing so, motivation for the task may be enhanced along with an increase in the adherence to treatment. Although this guide is oriented to the usually chosen age range of 6 to 18 years, generating different appearances depending on the patient’s age would likely be a good approach to treat ADHD during an individual’s growth and development. The same mechanics could even be used for adults by adopting more mature and sophisticated themes.

A serious video game also has the following requirements: (1) easy to understand, (2) require a minimal cognitive load (baseline), and (3) gradual level of difficulty. The baseline can be designed as an in-game tutorial. It is important to ensure that the person has understood the task and how to perform it. All of these elements were examined in the previously mentioned usability study for TSTM [22]. The aforementioned user-centered model [37] can help to adjust design guidelines.

#### Keeping a Patient Motivated With Serious Video Games

Sampayo-Vargas et al [41] stressed that in order to make educational computer games intrinsically motivational, it is critical to provide an optimal level of challenge. The difficulty curve should be neither too boring nor too frustrating. Users play the game and try to learn the correct pattern to follow to pass the given task and master it. Initially, they are entertained; however, when the game is no longer a challenge, users tend to become bored and give up [42]. For people with ADHD, novel challenges may improve adherence to treatment by increasing motivation. Furthermore, the game must automatically adjust to the individual’s performance level [37,41]. Games such as EndeavorRx [38] change the level of difficulty until the player is performing at an 80% rate of accuracy. Here, it is important to stress that artificial intelligence (AI) may help to personalize and update the needs of each individual in a specific context and time by adjusting at that precise moment (eg, type of game, intensity).

To avoid boredom, game design components should be included in any serious video game related to objects, mechanics, dynamics, and emotions [43]. “Juiciness” is a novel concept that refers to adding visual embellishments, sounds, and other types of nonfunctional elements, thus allowing developers to improve players’ experience in their games [29]. Juiciness should not affect the game mechanics but rather only focus on the extra elements (eg, menus, transitions, feedback), pursuing the development of a simple, minimalistic, intuitive, and easy-to-understand interface.

People with ADHD have limited self-perception and objectivity in task performance [4]. Therefore, providing simple and direct feedback as the player progresses consistently is a fundamental requirement in the design of the proposed serious video games [42]. To avoid too much frustration that may lead to abandoning the video game, points, badges, leaderboards, and other game components can be used. Moreover, introducing small doses of challenge could be helpful to improve frustration tolerance. Achieving engagement through the attractiveness of the task may benefit emotional dysregulation. Indeed, a recent review [44] concluded that regular, nonexcessive video game use can help to enhance emotional regulation in children. A good example is EmoGalaxy [45], which is a video game that improved emotional regulation in children diagnosed with ADHD.

Although video games are usually visually based, music and sounds are also fundamental elements that facilitate flow in video games alongside other elements. However, these elements should not be overwhelming, because people with ADHD are more likely to be distracted. Thus, audio-visual integration can be used to vary the intensity of emotional experience states [46]. To facilitate the sense of progress, missions can be completed. Performance can be quantified and made visible in changes in the user interface, counters, and score, among other elements [42]. This feedback can be provided by using as many senses as possible, including visual stimulus (ie, colors to show the level of performance), auditory stimulus (ie, sounds to point at a hit, error, task change), or nociceptive stimulus (ie, the vibration of the game controller can indicate that a mistake has been made).

#### Improving ADHD Symptoms

People with ADHD present broad clinical and cognitive variability [3]. Accordingly, any serious therapeutic intervention should ideally provide a tailored treatment targeting each patient’s specific difficulties and circumstances. In other words, serious video games should be developed within a personalized medicine framework [47].

Dovis et al [48] suggested that children with ADHD are less stimulated by reinforcement than typically developing children (likely due to a dopaminergic deficit), and therefore require higher amounts and frequencies of reward to perform optimally. These rewards should be more frequent at the beginning of the game (extrinsic motivation) and should decrease in frequency as the sessions progress, rewarding good performance with larger rewards (intrinsic motivation). However, people with ADHD may have delay aversion: a preference for small, immediate over larger, but delayed rewards [49]. Thus, it may be interesting to work on impulsivity through the reinforcement system [50]. Good task execution can also be rewarded through juicy embellishments and in-game achievements.

#### Avoiding Addiction

As previously discussed, people with ADHD may be more dependent on extrinsic motivation [51,52]. Children with ADHD are also more at risk of addiction to video games [13]. To avoid this, the focus should be on limiting the time spent playing. Furthermore, these time constraints may positively impact engagement by avoiding fatigue.

Research estimates that the average student's attention span is 10-20 minutes [53]; however, one of the symptoms of ADHD is sustained attention. Children with ADHD frequently experience hyper focus (the experience of focusing on something to the extent that all other stimuli are almost completely excluded) when playing video games [54]. Despite this, hyper focus cannot ensure that patients will continue to receive benefits, as illustrated in theories of deliberate practice [55].

Consequently, we have to reach a compromise between reinforcing the patient's attention beyond their own attention span and reducing gaming time to avoid addiction. Following the state of the art [15-17], treatments of 20-40 minutes 4-5 days a week seem to achieve more positive effects.

King et al [56] proposed five characteristics and their potential for game abuse: social features, manipulation and control features, narrative and identity features, reward and punishment features, and presentation features (see Table 1).

**Table 1:** Game characteristics and potential for game abuse (based on King et al [56]).

Game feature	Potential for game abuse
Social features	Do not play a relevant role in serious games, because they tend to be individually played
Control features	Need to master game controls
Resource management	Keeping resources above a minimum amount
Reward/punishment	Bonus content or additional resources associated with outstanding performance
Narrative and identity	Excess of immersion, excess of attachment to player’s avatar

Sessions should last approximately 20-30 minutes depending on the patient’s age. We recommend dividing the session into a variety of tasks [52]. Every 10 minutes, there can be a change of task (greater variability) or short breaks [4]. Furthermore, it is important to plan a sufficient number of sessions to consolidate learning. For instance, each TSTM training session lasts 25-30 minutes divided into three blocks. Two blocks of 10 minutes each are used for training with two game mechanics (Smasher, Kuburi, Tekateki, or Enigma), along with a third block of 5 minutes of chess-based game mechanics. The sessions are counterbalanced, randomizing the order of the blocks to avoid being repetitive or boring (see [23]).

#### Game Mechanics

According to Koster’s [57] definition in the Theory of Fun of Game Design, game mechanics are “rule-based systems/simulations that facilitate and encourage the user to explore and learn the properties of their possibility space through the use of feedback mechanisms.”

In a serious video game, game mechanics are the main intervention components through which patients acquire metacognitive strategies and skills to improve their cognitive abilities [18]. Game mechanics promote learning and repetition of the task, which can help to establish generalization.

Analyzing the serious video games included in several recent reviews [15-17] led us to the conclusion that almost all current ADHD treatments are structured in a compilation of mini games that generally tend to be similar to a regular video game (with respect to narrative, lore, esthetics, etc). In contrast, serious games for ADHD assessment are rarely more than one mini game, inspired by single tests. This is in keeping with a general recommendation of developing short and discrete tasks for people with ADHD, among others [43]. The use of mini games provides a clear advantage: the combination of mini games helps to personalize treatment for each patient by being able to distinguish which cognitive processes require more training.

Mini games can be created by adapting a cognitive task using game-up and mapping techniques [43] or from scratch. To attract attention, we can introduce design strategies such as gamifying the cognitive task stimuli to try to produce positive sensations. However, this process is specific to the disorder for which the treatment is intended. Khaleghi et al [43] proposed following the Objects, Mechanics, Dynamics, Emotions (OMDE) design guidelines. When designing game mechanics, it is critical to validate the cognitive aspects of the game. For example, in people with ADHD, it is necessary to control distractions, the cognitive load, and the time spent. Indeed, there are three main cognitive aspects that affect people with ADHD: attention, executive functions, and daily skills.

Inattention is one of the core symptoms of ADHD [2]. In traditional attention-training tasks, children with ADHD perform worse than typically developing children [52]. New mini games can be created to treat different types of attention. Additionally, ADHD presents great comorbidity with executive dysfunction [3]. Some games can help to train executive functions such as inhibition control, working memory, planning, or reasoning.

Lastly, one of the major criticisms of the use of serious video games is the lack of ecological validity [39,40]. Thus, proposed video games should induce the transfer of cognitive skills to domains of daily life functioning (transferability). Transferability is based in the improvement of different executive functions, which may help with scholarly development and motivation. Cognitive training is effective if the patient applies it in their daily life. For example, children with ADHD show impairments in social skills, which can have serious long-term consequences. Games that explore cooperation skills, such as “Space Club” [39], may improve social benefits. For example, TSTM incorporated chess mechanics, because chess can improve math performance, thus demonstrating transference into the educational domain [21].

### Exploration

The exploration stage of the IDEAL-Games framework [19] ensures that the video game meets the therapeutic objectives by testing the game’s feasibility, usability, reliability, and validity using proof-of-concept and pilot studies [39,43]. If not satisfactory, the development team can go back to the design phase, and revise the objectives, theoretical model, target variables, and players’ experience [18]. This stage should verify that the serious video game is ready (or not) for testing effectiveness. However, before testing the effectiveness, it is critical to verify that users enjoy the game [18]. A video game could be effective for treating ADHD, but will not be useful if patients do not use it because it is boring. Thus, feedback from patients may help to redesign certain elements. Furthermore, proof of concept, usability, and validity research studies help to detect bugs that may interfere with the following stages and to identify design errors, which would mean going back to the design stage and avoid conducting clinical studies that are destined to fail.

In the video game TSTM, we used UCD methodology [22,37] to follow precision gaming features, difficulty parameters, and other elements highlighted above. Feasibility was studied by conducting a market survey with professionals. The usability study of TSTM was assessed in people with ADHD to verify that the game was usable, of interest to the target group, easy to understand, and without side effects [22]. The reliability analysis allowed exploring the permanence of the measure over time (test-retest) or between raters (interrater), the consistency of the game (eg, with difficulty levels), or to determine whether two versions of the game (with and without VR) have similar results (parallel-forms reliability). In terms of validity, TSTM was considered to have content validity from the very beginning. For ecological validity, we monitored the transferability.

### Assessment of Effectiveness

Assessment is critical to determine the effectiveness of a serious video game [40]. Although research on health-based games has increased, the scientific basis remains too poor for a generalized recommendation [19]. High-quality studies (ie, randomized clinical trials) with sufficient sample size and time for the intervention are still warranted [43]. The key methodological limitations frequently encountered are inadequate controls, sustainability of training, and measures of cognitive function or inappropriate behavior [40]. Accordingly, we conducted a randomized trial with TSTM [23,58]. The patients’ self-reports demonstrated significant improvements in emotional regulation and adaptation to the social context (positive impression and interpersonal scale); 96% of the patients reported liking the experience of the clinical trial with the video game and having perceived a sense of improvement in all the cognitive abilities worked on (especially working memory and impulse control). The face validity also seemed to show that this treatment is more motivating owing to lower dropouts [58].

### Long-Term Assessment

As mentioned above, studies sometimes present difficulties in demonstrating transfer and ecological validity. Although some studies demonstrated that playing video games for 16 hours already produce certain changes in the brain [59], it is essential to understand the aspects of the game that are necessary for this to occur, and for how long these positive effects last. Monitoring the long-term effect of brand-new treatments such as video games is imperative, even if their short-term benefit has been clearly established.

## Discussion

### Summary

This guide aims to provide a roadmap for video game developers and researchers to build a serious video game that is fun, motivating, and effective in treating ADHD, while avoiding addictive properties. Following the IDEAL-Games proposal [19] and suggestions from Duncan et al [18] and Baranowski [20], among others, the proposed guide tries to bring together current knowledge on this topic. We have followed the order of the IDEAL process to develop the most important sections of this guide. We used our own experience in developing and testing TSTM, a serious video game specifically designed to treat ADHD. Both this guide and TSTM are based on information derived from game design methodologies, contributions from clinicians’ expertise, designers’ experience, children’s interests, and family feedback. TSTM usability results are good [22].

### Strengths and Limitations

The use of video games for treating ADHD has many advantages: (1) it is cheap, comfortable, flexible, and cost-efficient (eg, the time saved by avoiding travel alone would also indirectly benefit the environment); (2) the use of novel platforms (ie, VR/alternate reality) may improve not only adherence but also the clinical effects if transferability is demonstrated; and (3) the incorporation of AI may offer more personalized treatments for each patient.

Although serious video games promise to be an effective and interesting treatment platform, several limitations should be mentioned. For instance, serious video games must be tailored to the singularity of every patient (personalized medicine). We must also consider the possibility of some conditions such as motor difficulties, visual impairments, dyslexia, or color blindness, among others that may compromise effectiveness, and introduce adaptations if necessary. In addition, patients may be unfamiliar with technological platforms. Accordingly, a gradual approach is recommended. Furthermore, technological platforms could have side effects [22]. For example, VR can cause dizziness or discomfort in some users. Indeed, VR technology is not recommended in people with epilepsy or children aged 12 years or below. Another limitation is addiction [13]; thus, monitoring this risk is important [51]. Finally, follow-up studies are mandatory before serious video games can be incorporated within the multimodal treatment of ADHD.

### Conclusion and Future Directions

We hope that this guide assists the development of serious video games for ADHD. We stress the relevance of having a multidisciplinary team to balance clinical and technical skills [31]. Furthermore, finding a balance between enjoyment and addiction is mandatory. Moreover, new AI techniques may assist in creating tailored cognitive training schedules for each single patient. A virtual tutor may also help children to adhere to treatment by providing positive reinforcement while being monitored by therapists in parallel.

Despite the lack of studies comparing the effectiveness of commercial and serious video games on ADHD, we consider that serious video games have several advantages such as the specific design for symptoms and defective executive functions, although preventing the development of an addiction to the video game is essential.

Finally, evidence-based video serious games could help to complement multimodal treatment. Currently, the diagnosis of ADHD is based on clinical criteria, objective tests, and the subjective view of caregivers and teachers. However, recent studies suggest that video games may also assist in improving ADHD diagnoses. Future actions such as incorporating new technologies (ie, eye tracking, body trackers, brain-computer interfaces) while using a serious video game may help to improve the current subjective diagnoses of ADHD.

## Abbreviations

ADHD: attention deficit hyperactivity disorder
AI: artificial intelligence
CBT: cognitive behavioral therapy
OMDE: Objects, Mechanics, Dynamics, Emotions
TSTM: The Secret Trail of Moon
UCD: user-centered design
VR: virtual reality
